# Miscellany

**Published:** 1911-10

**Authors:** 


### MISCELLANY

---

### Thanks and an Explanation

We are duly appreciative of the many kind messages received from readers of the Journal, advertisers, and editors of other journals and for the cordial spirit of coöperation shown by all. With one exception, that of a firm which does not deal directly with physicians and which had carried an advertisement solely out of personal regard for our lamented predecessor, every regular advertising contract has been continued or renewed, several new ones have been added and several others are promised for the near future. With no time for a systematic canvass, over fifty new local subscribers have been secured, leaving less than fifty regular physicians, in actual practice, to be secured in Buffalo. Increased support has also been received from other cities and villages in our territory and we have been particularly gratified at receiving orders from several distant states, South Africa and South America.

It is only fair to state that the change of management was consummated after the September issue was partly printed—although the present editor had assisted somewhat with the two preceeding issues—and that the compliments received are very largely due to various writers, some of whom have modestly objected even to the initialing of their contributions and from all of whom, we hope to hear later.

We shall appreciate criticism and advice but it is self-evident that it will take a little time to get everyhing running smoothly.

### Parcels Post

HONORABLE JONATHAN BOURNE, JR., of Oregon, in the United States Senate, June 23, 1911, introduced the following bill (S. 2873):

*Be it enacted by the Senate and House of Representatives of the United States of America in Congress assembled,* That from and after the passage of this act no higher postage rate shall be charged for the transmission of mail entirely within the United States or its possessions than is charged for transmission of mail partly within and partly without the United States or its possessions. The Postmaster General is hereby authorised and required to establish and enforce rules and regulations which will give the people of the United States rights and privileges in the use of the United States mails as liberal as the rights and privileges the United States accords to the people of the most-favored nation.

Mr. Bourne commented as follows:

"It may seem strange to Members of the Senate that there should be a possibility of legislation giving American citizens privileges in the United States mails between themselves equal with those enjoyed by residents of this country in transaction of business with residents of foreign countries. The facts are these: Within the United States the rate of postage on fourth-class matter is 16 cents a pound, with a limit of 4 pounds. The United States is party to a treaty under which residents of 29 foreign countries may send fourth-class matter through the United States mails at 12 cents a pound, with a limit of 11 pounds. In other words, a man may send an 11-pound package from San Francisco to Rome, Italy, at 12 cents a pound, but if he wishes to send the same articles to New York he must divide them into packages of not to exceed 4 pounds each and pay 16 cents a pound."

THE Scottish Education Department advises disinfection of books by soaking sheets of blotting paper in 1:200 izal solution, placing each sheet between each two leaves, covering with a waterproof sheet for 24 hours and then gently removing the blotting paper when, if the soaking has not been too great, the book will be found uninjured. This process recalls the Frenchman's flea powder, consisting of powdered brick in attractive wrappers. To dissatisfied purchasers, he explained that the method of use was to hold the flea firmly in one hand and pour the powder down his throat till he choked to death. Except for books of exceptional value, we recommend that a more economic method would be to burn the book and buy a new one.

---

THE increase in cancer, diabetes, chronic interstitial nephritis and degenerational diseases generally, shown by almost all mortality statistics is viewed with alarm by one school of hygienists, while the opposite school, considers it rather a favorable indication. This paradox is explained as follows: It is true that cancer and degenerational diseases figure more largely in death reports than formerly but, on the other hand, the total mortality rate has markedly diminished. So too have the reports assigning death to unsatisfactory and indefinite terms such as "old age," "debility," "natural causes," etc. In other words, diagnosis is a more exact science, and the apparent increase in the diseases mentioned is not entirely genuine but due to more frequent detection of occult cases. Then too, there has been, in the past twenty years, an enormous saving of life during infancy, and, in adults, from tuberculosis, typhoid and miscellaneous infections. It is still too early to have any considerable increase of what may be termed degenerational material, from the wholesale saving of babies' lives, but quite time to expect that the hundred odd persons, per hundred thousand population, who have *not* died of typhoid in any one year 15 to 25 years ago, will now have to die of something else more appropriate to their increased age.

The Equitable Life Assurance Company has recently established a Department of Conservation (of human life), under the able management of E. A. Rittenhouse. This Department publishes an interesting paper called the *Human Factor.* Thanks to Mr. Rittenhouse, we are able to reproduce from it, the accompanying graphic illustration of the reciprocal relations of tuberculosis and degenerational diseases.

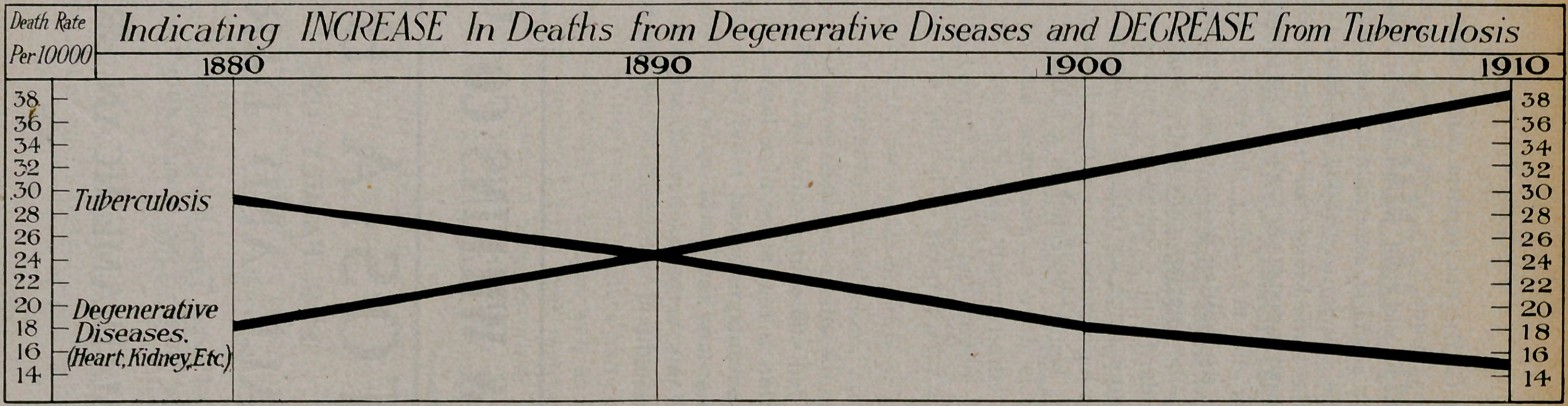

Death Rate Per 10000

Indicating INCREASE In Deaths from Degenerative Diseases and DECREASE from Tuberculosis

1880

1890

1900

1910

38

36

34

32

30

28

26

24

22

20

18

16

14

Tuberculosis

Degenerative Diseases. (Heart, Kidney, Etc.)

Major William G. Bissell reports colon bacilli in four brands of table water examined. In 1908-9, we attended a case of typhoid in a patient who had drunk nothing but one brand of table water, with a single exception of tap water, for several weeks prior to the attack. At about the same time, many thousand gallons of the same brand had been emptied out in another city because of the finding of colon bacilli but the producers claimed that this did not affect the supply for other places. Just why, unless the bottles were filled from local sources, we do not know. Some one has called attention that the vituperation of the Standard Oil Company is undeserved when water is sold at the same price as refined kerosene. If table water at an absurdedly high price is not free from gross contamination, the public is being subjected to a fraud of the worst kind. Until something is done to guarantee against impure bottled water, the plain citizen might as well drink ordinary tap water and he can save money and protect himself effectually against infection by the simple process of boiling and cooling.

---

An old friend of the editor's wishes to retire from practice and offers his home and office, in a town of 10,000 for sale or lease. The town is within a few miles of a large city and offers exceptional social advantages while, as to the practice, our friend expects to travel around the world on his profits. It is a curious coincidence that this very practical illustration of one of our editorials was brought to our notice while the latter was being printed.

